# Supplementary material for: Membrane topography and the overestimation of protein clustering in single molecule localisation microscopy – identification and correction
Source: Commun Biol. 2024 Jun 29;7:791. doi: 10.1038/s42003-024-06472-3 (PMC11217499; doi:10.1038/s42003-024-06472-3)
Supplement: Supplementary file 2 — Supplementary Information [file 42003_2024_6472_MOESM2_ESM.pdf]

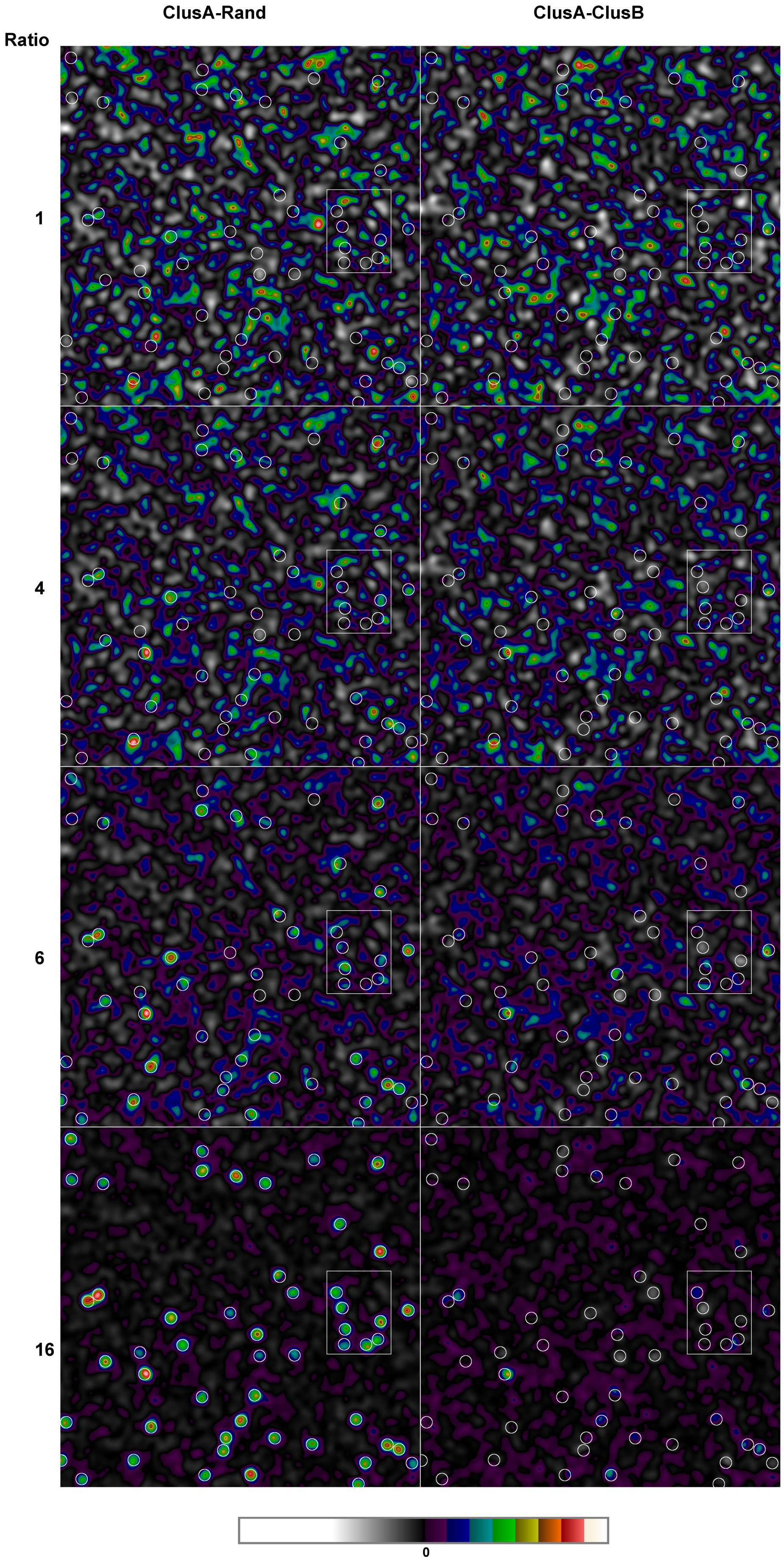

ClusA-Rand
ClusA-ClusB
Ratio
1
4
6
16
0

Supplementary Figure 1 | Simulated distributions of protein molecules after subtraction of a membrane marker. Simulated 2048x2048 pixel datasets with a constant number of localizations of a protein marker (ClusA) split between the designated clustered (marked circles, covering 4% of the total area) and non-clustered areas (96%). The localization density difference (ratio) between the clustered and non-clustered areas was incrementally increased while keeping the total number of localizations constant. The clusters were randomly positioned within the borders and without any overlap. Note the ratio of 1 means that the image does not contain areas of deliberate localisation enrichment, only those arising randomly. The numbers are the ratios of the localization density between the clustered and non-clustered areas. The datasets are displayed after Gaussian filtering (sigma 20 pixels) and removing a guard area. Rand is a membrane molecule randomly distributed in a flat and smooth membrane. ClusB is a membrane marker partitioning at the same ratios to the cluster areas as the protein marker (ClusA). Smaller cluster-rich parts of the datasets (marked with squares) are shown in Figure 2.

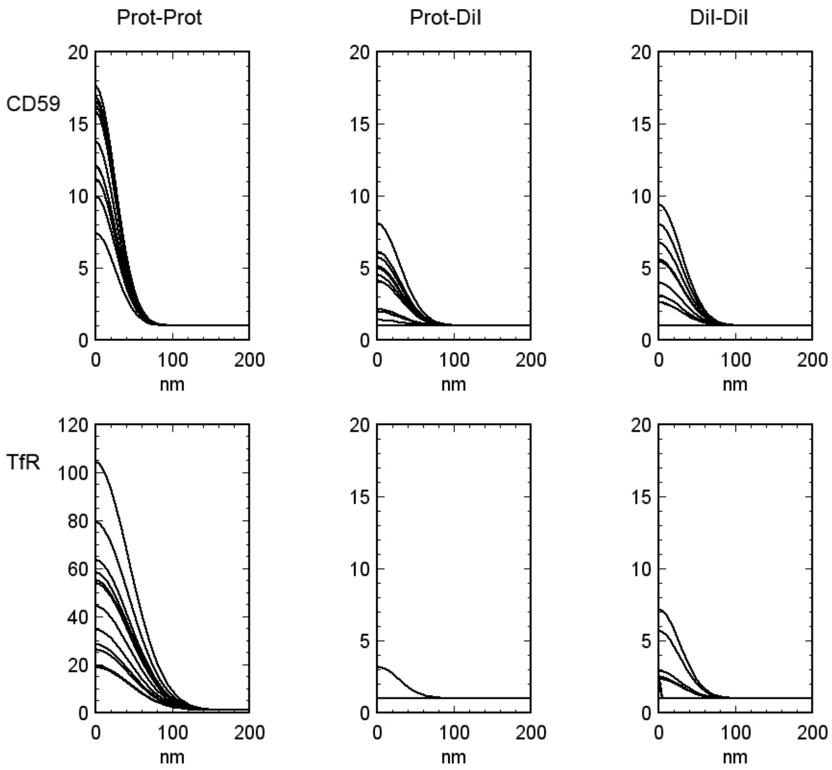

Supplementary Figure 2 | PC-PALM analysis identifies DiI-C12, CD59 and the TfR as genuinely clustered. PC-PALM analysis, designed to eliminate the clustering contribution of repeated detections of the same fluorophores, was applied to the live cell SMLM-data displayed in Figure 3. 10 out of 25 snapshots from the entire dataset are displayed. For the TfR-DiI combination, nine out of the ten datasets form a straight line at the value of one.

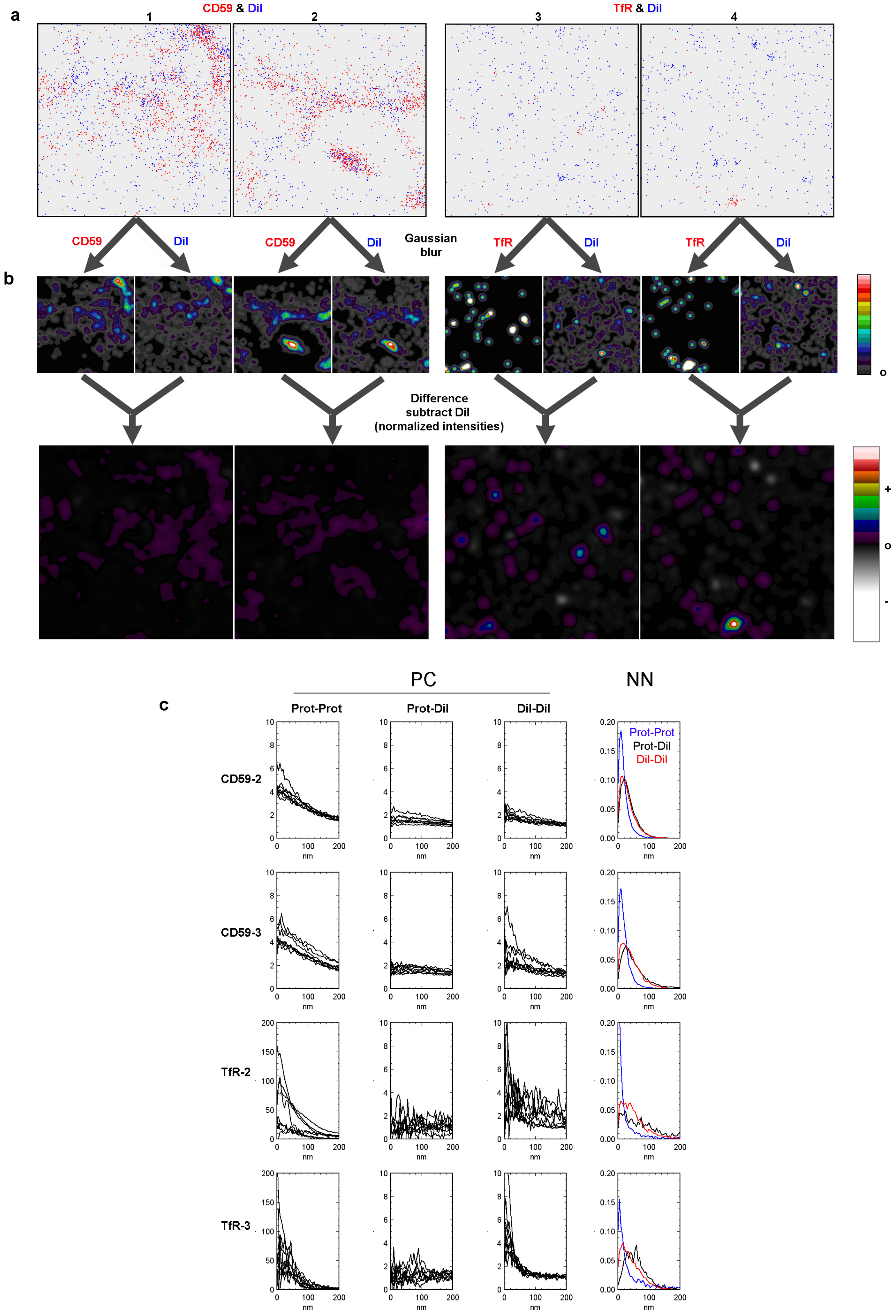

Supplementary Figure 3 | Live cell dual colour SMLM with a protein (CD59 or TfR) and the membrane probe DiI to identify clusters caused by membrane topography variations. (**a**) Four 2.5x2.5 µm SMLM 7s snapshots of different cells stained with DiI and CD59 or the TfR

visualised with Alexa Fluor-647 conjugated antibodies. (**b**) Gaussian filter (sigma 8 nm) versions of the snapshots. All eight images were normalised to the same mean intensity and the DiI images subtracted from those of the proteins. The two difference images use the same intensity range without any contrast alteration and the intensity scale is symmetrical around 0 with intensities below 0 shown with a greyscale. (**c**) Nearest neighbour analysis and pair correlation analysis of the protein molecule to itself, to DiI and DiI-DiI. The NN analysis is from the entire dataset. For PC analysis the representative results from 10 out of 25 snapshots from the entire dataset are displayed. Note that five of the six PC graphs use the same y-axis range but the TfR prot-prot graph uses a much wider range.

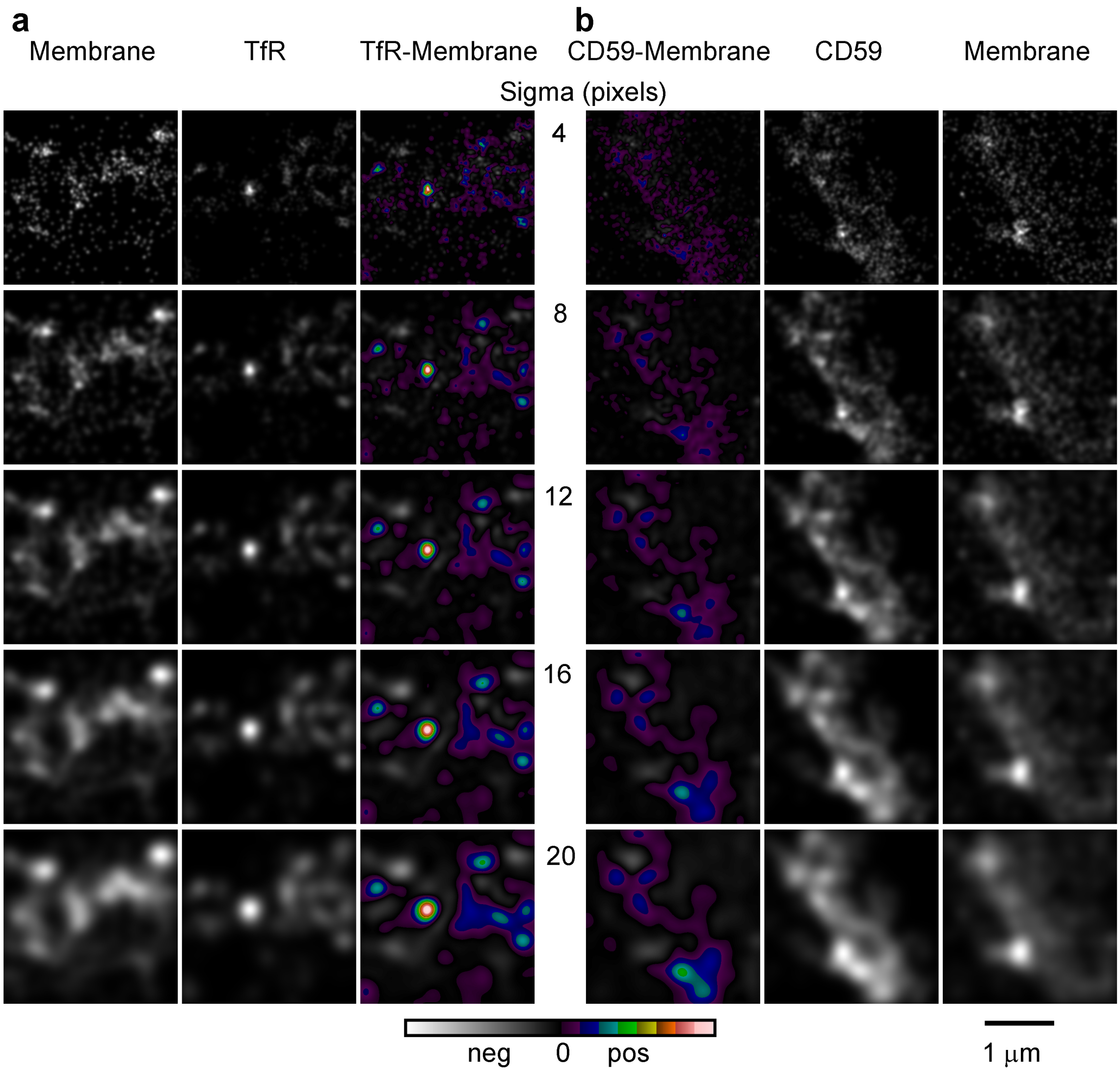

Supplementary Figure 4 | The effect of image smoothing on cluster identification. 2.5x2.5 μm SMLM 7s snapshots of (**a**) DiI and TfR and (**b**) CD59 and DiI the TfR (the same images as in Figure 3). The proteins were labelled with Alexa Fluor-647 conjugated antibodies and the membrane was identified using DiI-C12. The four images were normalised to the same mean intensity and subjected to smoothing with a Gaussian filter with different sigma (4, 8, 12, 16 and 20 pixels). The two protein and two membrane images are displayed in greyscale with the same intensity range. The centre two images in each row show the result of subtracting the respective DiI image from that of the relevant protein. The difference images use the same intensity range without any contrast alteration and the intensity scale is symmetrical around zero with negative intensities shown with a greyscale.

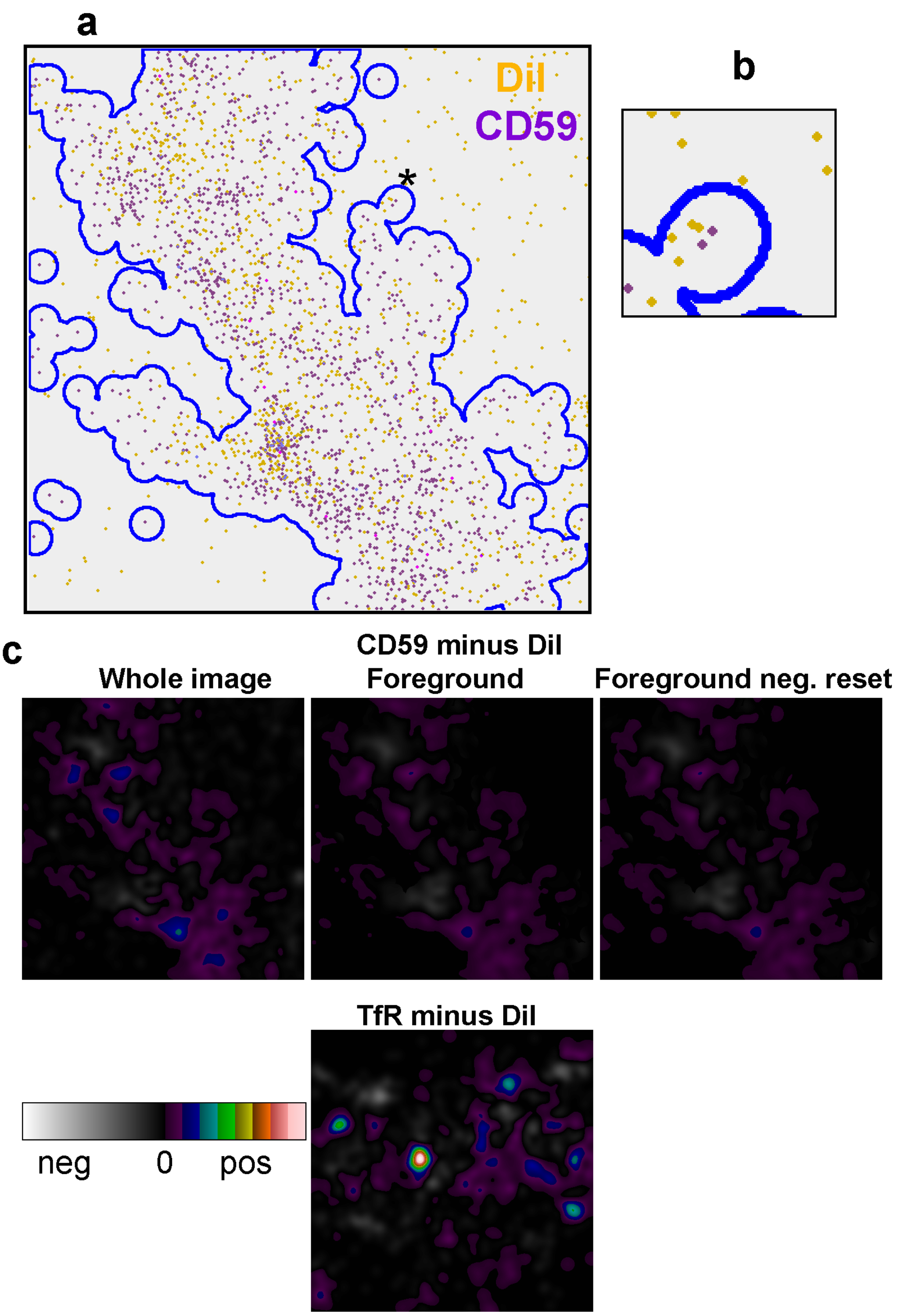

Supplementary Figure 5 | The effect of background subtraction on cluster appearance. (**a**) The background in the CD59/DiI dataset was defined as the area without CD59. (**b**) Magnification of the area marked with * in a. (**c**) Difference images of CD59 – DiI and TfR-DiI (the same datasets as in Figure 3 and Supplementary Figure 4). The datasets were subjected to smoothing with a Gaussian filter with sigma 8 pixels. The image pairs were then normalised to a common intensity mean, and the membrane marker image subtracted. For the CD59/DiI dataset, the mean DiI background was subtracted from the smoothed DiI image in the two images to the right. After background subtraction, the foreground area was used for

the normalisation. Top row: difference images of CD59-DiI. Top left: without background subtraction using the whole image. Top centre: using the foreground area including negative intensity value pixels. Top right: using the foreground with the negative intensity value pixels set to zero. Bottom row: TfR-DiI. The images are displayed in greyscale with the same intensity range and the intensity scale is symmetrical around zero with negative intensities shown with a greyscale.

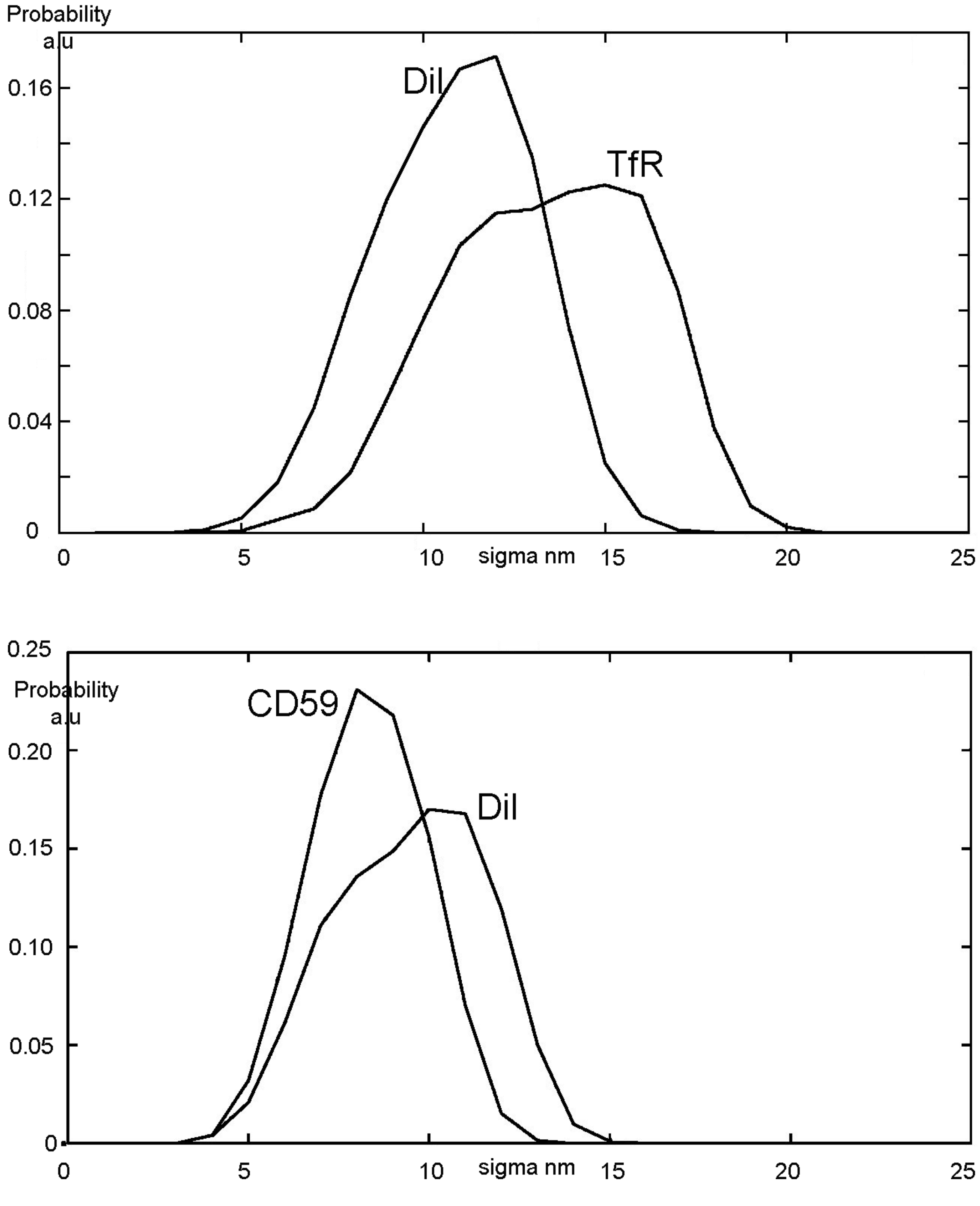

Supplementary Figure 6 | The localisation precision for DiI-C12, CD59 and the TfR in live cell SMLM. (**a**) Sigma for the localisation precision for the DiI/TfR and (**b**) CD59/DiI datasets. All localisation from all datasets were included in the graphs.
