# Supplementary material for: Membrane topography and the overestimation of protein clustering in single molecule localisation microscopy – identification and correction
Source: Commun Biol. 2024 Jun 29;7:791. doi: 10.1038/s42003-024-06472-3 (PMC11217499; doi:10.1038/s42003-024-06472-3)
Supplement: Supplementary file 3 — Description of Additional Supplementary Files [file 42003_2024_6472_MOESM3_ESM.pdf]

Description of Additional Supplementary Files

File name: Supplementary Data 1

Description: The source data for the graph in Figure 2g.

File name: Supplementary Data 2

Description: The source data for Figure 3, 1-2 DiI-TfR 3-4 DiI-CD59.

File name: Supplementary Data 3

Description: The source data for the graph in Figure 4c, 1-2 adjacent, 3-4 random.
