# Supplementary figures and images for: Membrane topography and the overestimation of protein clustering in single molecule localisation microscopy – identification and correction
Source: Commun Biol. 2024 Jun 29;7:791. doi: 10.1038/s42003-024-06472-3 (PMC11217499; doi:10.1038/s42003-024-06472-3)

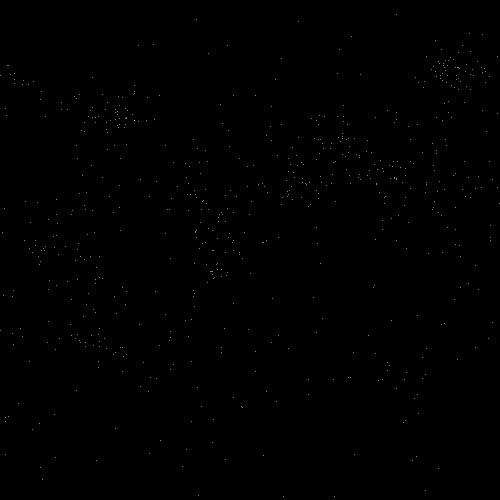

Supplement: Supplementary file 5 — Supplementary Data 2 [file 42003_2024_6472_MOESM5_ESM.tif]

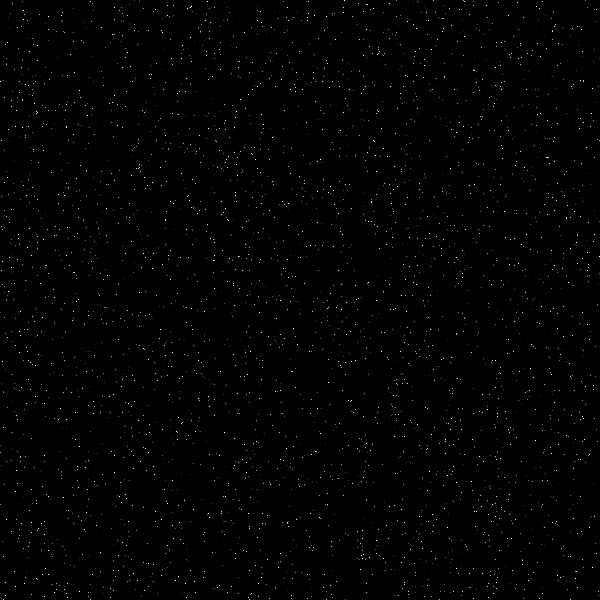

Supplement: Supplementary file 6 — Supplementary Data 3 [file 42003_2024_6472_MOESM6_ESM.tif]
