# Supplementary material for: Membrane topography and the overestimation of protein clustering in single molecule localisation microscopy – identification and correction
Source: Commun Biol. 2024 Jun 29;7:791. doi: 10.1038/s42003-024-06472-3 (PMC11217499; doi:10.1038/s42003-024-06472-3)
Supplement: Supplementary file 7 — Reporting summary [file 42003_2024_6472_MOESM7_ESM.pdf]

nature portfolio

Corresponding author(s): Ingela Parmryd

Last updated by author(s): Jun 15, 2024

Reporting Summary

Nature Portfolio wishes to improve the reproducibility of the work that we publish. This form provides structure for consistency and transparency in reporting. For further information on Nature Portfolio policies, see our Editorial Policies and the Editorial Policy Checklist.

Statistics

For all statistical analyses, confirm that the following items are present in the figure legend, table legend, main text, or Methods section.

n/a	Confirmed	
☐	☒	The exact sample size (n) for each experimental group/condition, given as a discrete number and unit of measurement
☐	☒	A statement on whether measurements were taken from distinct samples or whether the same sample was measured repeatedly
☐	☒	The statistical test(s) used AND whether they are one- or two-sided<br>*Only common tests should be described solely by name; describe more complex techniques in the Methods section.*
☒	☐	A description of all covariates tested
☒	☐	A description of any assumptions or corrections, such as tests of normality and adjustment for multiple comparisons
☒	☐	A full description of the statistical parameters including central tendency (e.g. means) or other basic estimates (e.g. regression coefficient) AND variation (e.g. standard deviation) or associated estimates of uncertainty (e.g. confidence intervals)
☐	☒	For null hypothesis testing, the test statistic (e.g. F, t, r) with confidence intervals, effect sizes, degrees of freedom and P value noted<br>*Give P values as exact values whenever suitable.*
☒	☐	For Bayesian analysis, information on the choice of priors and Markov chain Monte Carlo settings
☒	☐	For hierarchical and complex designs, identification of the appropriate level for tests and full reporting of outcomes
☒	☐	Estimates of effect sizes (e.g. Cohen's d, Pearson's r), indicating how they were calculated

Our web collection on statistics for biologists contains articles on many of the points above.

Software and code

Policy information about availability of computer code

Data collection	The simulated datasets were generated in ImageJ. The ImageJ macros used to generate and analyse the data are available at https://github.com/Parmryd/Membrane_Topography_and_Protein_Clustering
Data analysis	STORM image localization was performed using SMLocalizer.<br>Skewness was analysed using the skewness function type 3 in the package e1071 version 1.6-8 in R statistical software version 3.4.3.

For manuscripts utilizing custom algorithms or software that are central to the research but not yet described in published literature, software must be made available to editors and reviewers. We strongly encourage code deposition in a community repository (e.g. GitHub). See the Nature Portfolio guidelines for submitting code & software for further information.

Data

Policy information about availability of data

All manuscripts must include a data availability statement. This statement should provide the following information, where applicable:

- Accession codes, unique identifiers, or web links for publicly available datasets
- A description of any restrictions on data availability
- For clinical datasets or third party data, please ensure that the statement adheres to our policy

The simulated and SMLM datasets used in this study are provided as Supplementary data.

Research involving human participants, their data, or biological material

Policy information about studies with human participants or human data. See also policy information about sex, gender (identity/presentation), and sexual orientation and race, ethnicity and racism.

Reporting on sex and gender	*Use the terms sex (biological attribute) and gender (shaped by social and cultural circumstances) carefully in order to avoid confusing both terms. Indicate if findings apply to only one sex or gender; describe whether sex and gender were considered in study design; whether sex and/or gender was determined based on self-reporting or assigned and methods used. Provide in the source data disaggregated sex and gender data, where this information has been collected, and if consent has been obtained for sharing of individual-level data; provide overall numbers in this Reporting Summary. Please state if this information has not been collected. Report sex- and gender-based analyses where performed, justify reasons for lack of sex- and gender-based analysis.*
Reporting on race, ethnicity, or other socially relevant groupings	*Please specify the socially constructed or socially relevant categorization variable(s) used in your manuscript and explain why they were used. Please note that such variables should not be used as proxies for other socially constructed/relevant variables (for example, race or ethnicity should not be used as a proxy for socioeconomic status). Provide clear definitions of the relevant terms used, how they were provided (by the participants/respondents, the researchers, or third parties), and the method(s) used to classify people into the different categories (e.g. self-report, census or administrative data, social media data, etc.) Please provide details about how you controlled for confounding variables in your analyses.*
Population characteristics	*Describe the covariate-relevant population characteristics of the human research participants (e.g. age, genotypic information, past and current diagnosis and treatment categories). If you filled out the behavioural & social sciences study design questions and have nothing to add here, write "See above."*
Recruitment	*Describe how participants were recruited. Outline any potential self-selection bias or other biases that may be present and how these are likely to impact results.*
Ethics oversight	*Identify the organization(s) that approved the study protocol.*

Note that full information on the approval of the study protocol must also be provided in the manuscript.

Field-specific reporting

Please select the one below that is the best fit for your research. If you are not sure, read the appropriate sections before making your selection.

- [x] Life sciences
- [ ] Behavioural & social sciences
- [ ] Ecological, evolutionary & environmental sciences

For a reference copy of the document with all sections, see nature.com/documents/nr-reporting-summary-flat.pdf

Life sciences study design

All studies must disclose on these points even when the disclosure is negative.

Sample size	The imaging of the two protein probes together with the membrane marker was performed on the same day at two occasions (TfR N=11, CD59 N=7 with 25 snapshots/dataset).
Data exclusions	For visualisation purposes 10/25 representative snapshots for the pair correlations analysis are displayed in Figure 3 and Supplementary Figure 3. No exclusion criteria applied.
Replication	The findings from cells reported in Figure 3 are replicated with more cells in Supplementary Figure 3.
Randomization	The first encountered well-stained cell without signs of damage was used for data collection. This process was repeated at random locations on each coverslip until DiI internalization became apparent.
Blinding	Blinding was not considered necessary since the cell selection for imaging was randomized and all analysis performed with the datasets standardised so that they were identical for each cell.

Reporting for specific materials, systems and methods

We require information from authors about some types of materials, experimental systems and methods used in many studies. Here, indicate whether each material, system or method listed is relevant to your study. If you are not sure if a list item applies to your research, read the appropriate section before selecting a response.

Materials & experimental systems

n/a	Involved in the study
☐	☒ Antibodies
☐	☒ Eukaryotic cell lines
☒	☐ Palaeontology and archaeology
☒	☐ Animals and other organisms
☒	☐ Clinical data
☒	☐ Dual use research of concern
☒	☐ Plants

Methods

n/a	Involved in the study
☒	☐ ChIP-seq
☒	☐ Flow cytometry
☒	☐ MRI-based neuroimaging

Antibodies

Antibodies used

Anti-CD59 Alexa Fluor-647 (clone MEM-43, Catalogue number A6-233-T025, lot 528981) and anti-TfR Alexa Fluor-647 (clone MEM-75, Catalogue number A6-235-T025, lot 528982) from EXBIO Praha, Vestec, Czech Republic.

Validation

From manufacturers site:
The antibody MEM-43 reacts with well defined epitope (W40, R-53) on CD59 (Protectin), an 18-20 kDa glycosylphosphatidylinositol (GPI)-anchored glycoprotein expressed on the surface of all hematopoietic cells; it is widely present on cells in all tissues. This antibody does not compete with MEM-43/5, and has blocking activity.

The antibody MEM-75 reacts with an extracellular epitope of CD71 antigen (transferrin receptor), a 95 kDa type II homodimeric transmembrane glycoprotein expressed on activated B and T lymphocytes, macrophages and erythroid precursors; it is lost on resting blood leukocytes. The antibody MEM-75 does not block binding of transferrin to the receptor.

Purified antibody is conjugated with Alexa Fluor® 647 NHS ester under optimum conditions and unconjugated antibody and free fluorochrome are removed by size-exclusion chromatography.

Eukaryotic cell lines

Policy information about cell lines and Sex and Gender in Research

Cell line source(s)

HT29 human colon adenocarcinoma cells from ATCC.

Authentication

Cell line identity was provided by ATCC.

Mycoplasma contamination

The cultures tested negative for mycoplasma infection.

Commonly misidentified lines
(See ICLAC register)

Name any commonly misidentified cell lines used in the study and provide a rationale for their use.

Plants

Seed stocks

Report on the source of all seed stocks or other plant material used. If applicable, state the seed stock centre and catalogue number. If plant specimens were collected from the field, describe the collection location, date and sampling procedures.

Novel plant genotypes

Describe the methods by which all novel plant genotypes were produced. This includes those generated by transgenic approaches, gene editing, chemical/radiation-based mutagenesis and hybridization. For transgenic lines, describe the transformation method, the number of independent lines analyzed and the generation upon which experiments were performed. For gene-edited lines, describe the editor used, the endogenous sequence targeted for editing, the targeting guide RNA sequence (if applicable) and how the editor was applied.

Authentication

Describe any authentication procedures for each seed stock used or novel genotype generated. Describe any experiments used to assess the effect of a mutation and, where applicable, how potential secondary effects (e.g. second site T-DNA insertions, mosiacism, off-target gene editing) were examined.
